# Erythema Nodosum and Tuberculosis

**Published:** 1910-02-12

**Authors:** 


### Dermatology.

### ERYTHEMA NODOSUM AND TUBERCULOSIS.

Erythema Nodosum is an affection characterised by the presence of red, tender, oval-shaped nodes, situated generally on the legs below the knees, with their long diameter in the long axis of the limb, and usually on the front aspect of the leg. The nodes may occur also, though less often, on the forearms. The onset and course of this affection are acute. It begins with a feeling of malaise, perhaps with shivering, pains in the joints and a little soreness of the throat. Then the tender nodes appear, and these, after remaining for a week or so, gradually disappear. They become first of all less red, then yellowish-brown like a fading bruise, and finally fade altogether. A second attack is rare. This affection has been attributed from time to time to different causes. Towards the middle of the last century it was regarded as of rheumatic origin. Then it gradually came to be regarded as an independent affection of an exanthematous nature probably due to an unknown specific micro-organism. Then it became the fashion to regard many diseases of unknown origin as toxic infections, auto-intoxications, or toxæmias, and the eruption of erythema nodosum was considered to be a symptomatic manifestation of various different toxins. Quite recently in France many observers have been applying the local tuberculin tests of Calmette, Pirquet, Moro and others to cases of erythema nodosum, with the result that a very large proportion of these cases have been found to give positive reactions, so that it is now claimed by these observers that erythema nodosum is in most cases an indication of tuberculosis. They admit that as a rule the presence of tuberculosis is not evident at the time, and is only revealed by the positive reaction of tuberculin, but many examples have been given in which tuberculous affections have developed subsequently. In a small proportion of cases of erythema nodosum there has been associated tuberculosis, but since the very large majority have manifested no symptoms of tuberculosis, the association has hitherto been regarded as accidental. Although further evidence is required before accepting the view that erythema nodosum is a toxi-tuberculous eruption, the possibility of a latent tuberculosis is worth bearing in mind in the presence of a case of erythema nodosum.

Erythema nodosum must, of course, not be confused with the affection known as erythema induratium scrofulosorum or Bazin's disease, in which the lesions occur mainly on the calves of the legs, are indolent and painless and of very slow development, and with a tendency to break down into ulcers. This latter affection is seen chiefly in young women who stand much, and, as is implied by the epithet "scrofulosorum," it has always been regarded as having associations with tuberculosis.

